# Blood Flow Restriction Training, Molecular Modulators, and Musculoskeletal Health: A Scoping Review and Translational Perspective

**DOI:** 10.3390/ijerph23050567

**Published:** 2026-04-28

**Authors:** Charlotte Georgia Anderson, Sarabjit Mastana

**Affiliations:** School of Sport, Exercise and Health Sciences, Loughborough University, Loughborough LE11 3TU, UK; charlottegeo88@gmail.com

**Keywords:** blood flow restriction, resistance training, genetic variation, muscle hypertrophy, strength, personalised exercise, musculoskeletal health

## Abstract

**Highlights:**

**Public health relevance—How does this work relate to a public health issue?**

- BFRT produces strength and hypertrophy with minimal mechanical load, making it suitable for ageing populations, those at risk of sarcopenia, and rehabilitation where high loads are contraindicated. Short sessions, modest equipment requirements, and adaptability across clinical, community, and sport settings support scalable exercise therapy.
- Inter-individual variability highlights the need for more personalised, effective public health exercise prescriptions.

**Public health significance—Why is this work of significance to public health?**

- BFRT promotes muscle adaptation through key molecular pathways associated with muscle growth and vascular function. Genotype-based BFRT studies/analyses are currently lacking but should be comprehensively explored for genetic architecture and possible clinical implementation.

**Public health implications—What are the key implications or messages for practitioners, policy makers and/or researchers in public health?**

- BFRT is suitable for load-intolerant patients and adults with individualised cuff pressure and standardised protocols.
- Adequately powered, genotype-stratified randomised controlled trials with standardised BFR parameters and integrated molecular, morphological, and functional endpoints in diverse populations are necessary.

**Abstract:**

Background: Blood flow restriction training (BFRT) is a low-load resistance training modality capable of inducing muscle hypertrophy and strength adaptations that are comparable to traditional high-load resistance training. Beyond athletic performance settings, BFRT has growing relevance for musculoskeletal health, rehabilitation and populations unable to tolerate high mechanical loads. However, substantial inter-individual variability in adaptive responses has been reported. Genetic and molecular factors may partly contribute to this variability and inform more individualised exercise strategies. Other intrinsic and extrinsic factors, including age, sex, training status, nutrition, and protocol-related differences, may also influence adaptive responses. Objective: This scoping review aimed to map available evidence on molecular modulators of adaptation to BFRT and to identify gaps in the literature regarding genetic influences on BFRT responses. Methods: A structured search of PubMed, Web of Science and Google Scholar was conducted till 1 February 2026. Experimental and quasi-experimental studies examining BFRT in relation to genetic polymorphisms, gene expression, and molecular signalling pathways associated with strength and hypertrophy outcomes were included. Primary outcomes were genetic and molecular factors relevant to BFRT adaptation, including genetic polymorphisms, gene expression, and molecular signalling markers. Secondary outcomes included muscle strength, hypertrophy, vascular responses, and related functional outcomes where reported. Study selection and data extraction were conducted according to PRISMA-ScR guidelines. The methodological quality of randomised controlled trials was assessed using the PEDro scale. This scoping review was registered retrospectively in the Open Science Framework on 17 March 2026, after completion of the literature search. Results: From an initial 47 records, only three studies (*n* = 3) met the inclusion criteria. The included studies reported molecular responses associated with BFRT, including downregulation of proteolytic genes, suppression of myostatin expression, and upregulation of angiogenic markers. Notably, no studies directly examined genetic polymorphism or genotype–BFRT interactions, highlighting a clear need for these studies in this field. Conclusions: This scoping review therefore identifies a critical evidence gap, with genotype-informed BFRT prescription remaining unsupported by the current literature. Limited evidence supports the possible role of BFRT in molecular responses associated with muscle adaptation. Future research should prioritise well-designed studies integrating both genetic and molecular analyses to better understand inter-individual variability in BFRT adaptations.

## 1. Introduction

Resistance training is widely considered as a cornerstone for improving muscular strength and hypertrophy [1]. Traditionally, high-load resistance training (HL-RT) has been considered the most effective method for inducing these adaptations. However, the substantial mechanical stress associated with HL-RT can increase fatigue and elevate the risk of musculoskeletal injuries [2]. Consequently, there is growing interest in alternative modalities that achieve comparable muscular adaptations while minimising mechanical strain [3,4].

Blood flow restriction training (BFRT), also referred to as occlusion training, involves applying external pressure via cuffs to proximal limbs during low-load resistance exercise (20–30% 1RM), creating a hypoxic and metabolically stressful environment [5,6,7]. BFRT may promote muscular adaptations through metabolic stress, including hypoxia, lactate accumulation and fast-twitch fibre recruitment, leading to activation of hypertrophic and angiogenic pathways [8,9,10]. This enables increases in muscle strength and hypertrophy with substantially lower mechanical load [11,12,13,14,15,16].

In healthy athletic populations, BFRT is particularly valuable due to its ability to induce adaptations while minimising mechanical stress on joints and connective tissue [17,18]. A meta-analysis [19] reported that low-load resistance training with BFR (<50% 1RM) produced greater or comparable gains in muscle strength and hypertrophy compared to traditional high-load resistance training (>70% 1RM). Similarly, ref. [20] reported that professional soccer players achieved comparable increases in thigh muscle size (+3.3%) using low-load BFRT (20–35% 1RM) and high-load training (70–85% 1RM). However, these findings should be interpreted cautiously due to methodological variability, short intervention durations and heterogeneous populations [17].

Beyond performance settings, resistance training responsiveness is also highly relevant to broader musculoskeletal health, ageing, and rehabilitation contexts. Variability in strength and hypertrophic adaptations may influence long-term functional capacity, injury resilience and quality of life. Understanding biological contributors to this variability, including genetic factors, may therefore have implications not only for athletic development but also for personalised exercise prescription in wider health settings.

### Possible/Potential Role of Genetic Factors in Exercise Response

Genetic variation plays a vital role in modulating individual responses to exercise, including increased hypertrophy, strength and overall athletic performance. Variants such as *ACTN3* R577X, *IGF-1* and *MSTN* have been identified as key contributors to these adaptations. While earlier research focused heavily on sprint and power outcomes, more recent studies have emphasised the broader importance of genes in resistance training and muscular development [21,22,23].

A meta-analysis of the *ACTN3* gene [23] reported that both the RR genotype and R allele are significantly overrepresented in strength and power athletes compared to endurance athletes and non-athletic controls. This association suggests that *ACTN3* R577X is a potential genetic marker for power performance; however, its influence on BFRT adaptations remains unclear.

*IGF-1* is a critical regulator of muscle growth, promoting protein synthesis, satellite cell activation and fibre hypertrophy [24]. A recent study [25] reported that the rs35767 (C-1245T) variant in the *IGF-1* promoter region is more common in athletes, therefore indicating a role in power, strength and endurance performance. This variant likely enhances *IGF-1* transcription, promoting greater adaptations to loading stimuli. However, its relevance to BFRT adaptations has not been directly established.

The *MSTN* gene encodes myostatin, which limits muscle growth. The K153R (rs1805086) variant reduces myostatin activity, enabling greater hypertrophy [26]. A 2022 meta-analysis [27] examined the *MSTN* K153R (rs1805086) polymorphism and found that strength-oriented athletes were about twice as likely to carry the R allele compared to non-athletes. This indicates a significant association between this genetic variant and an enhanced strength and muscle mass phenotype. In addition to genetic polymorphisms, acute molecular responses to exercise, such as gene expression changes and signalling pathway activation, may also contribute to variability in training adaptations; however, these represent transient responses rather than fixed genetic traits.

Given BFR’s reliance on metabolic stress and vascular adaptation, variants such as *NOS3* or *MCT1* may be particularly relevant due to their role in endothelial function and lactate metabolism [28,29].

Given the growing interest in BFRT and the increasing recognition of inter-individual variability in exercise adaptation, this scoping review aimed to map whether genetic polymorphisms and molecular factors associated with strength and hypertrophy responses have been investigated in the context of BFRT. A secondary aim was to summarise the molecular pathways reported in BFRT studies and to examine their relevance to personalised exercise prescription and musculoskeletal health.

Although several genetic variants are associated with strength, hypertrophy, and vascular traits, no studies have directly linked these variants to BFRT responses.

## 2. Materials and Methods

This scoping review was conducted and reported in accordance with PRISMA-ScR guidelines [30] to ensure transparency and methodological rigour. The review was registered retrospectively in the Open Science Framework (OSF; https://osf.io/hypt3/ (accessed on 17 March 2026)) on 17 March 2026, after completion of the literature search. This should be considered a methodological limitation. Given the emerging and limited evidence base, a qualitative synthesis approach was adopted, as a meta-analysis was not appropriate. A limited methodological quality appraisal was conducted for randomised controlled trials using the PEDro scale to provide contextual insight. Because the included evidence also comprised non-randomised and mechanistic studies, a formal risk-of-bias comparison across all designs was not undertaken.

The PICOS framework (Population, Intervention, Comparison, Outcome, and Study Design) was used to define the research question and establish eligibility criteria. Relevant studies were identified through structured electronic database searches conducted up to 1 February 2026. Studies were screened and selected according to predefined eligibility criteria. Accordingly, this review not only synthesises existing molecular evidence but also explicitly maps the absence of genetic polymorphism research within the BFRT literature.

All stages of the review process, including screening, data extraction and appraisal, were performed and independently verified by two researchers to minimise bias and enhance the reliability of the findings.

### 2.1. Search Strategy

The search was restricted to studies published in English and within the last 15 years. This timeframe was chosen to reflect the relatively recent uptake and methodological standardisation of BFRT, as well as contemporary molecular and genetic techniques used in exercise physiology. To achieve this, the electronic databases of PubMed, Web of Science and Google Scholar were utilised. Due to the broad scope of Google Scholar, screening was limited to the first 200 results (approximately 20 pages), sorted by relevance to ensure feasibility and the relevance of included studies. Duplicate records identified across databases were removed using Covidence software (https://www.covidence.org/ (accessed on 17 March 2026)). The selection of these databases was informed by methodological research indicating that no single database provides complete coverage of the literature and that combining multiple databases is necessary to maximise retrieval of relevant studies. Because no single database provides complete coverage of the literature, PubMed, Web of Science, and Google Scholar were searched to improve retrieval breadth while maintaining feasibility.

These databases were systematically searched using carefully selected keywords and Boolean operators. Terms related to blood flow restriction, such as ‘BFR training’, ‘blood flow restriction training’ and ‘vascular occlusion’, were combined with keywords about muscular strength and hypertrophy, including ‘strength’, ‘muscle strength’, ‘muscle hypertrophy’, ‘muscle growth’, ‘muscle size’ and ‘muscle mass’. In addition, genetic factors were incorporated using terms such as ‘genetic’, ‘genetics’, ‘gene’, ‘polymorphism’, ‘genotype’, ‘genetic variation’ and ‘genetic predisposition’. Finally, to focus the search on the target population, terms like ‘athletic’, ‘healthy’, ‘sportspeople’ and ‘sportsperson’ were included. The timeframe was selected to ensure that the included studies reflect contemporary BFRT methodologies, as well as current advances in molecular and genetic research techniques relevant to exercise physiology. The final searches were conducted in February 2026. In addition to database searching, citation searching of included studies was undertaken to identify any additional relevant articles. The full search strategies, including complete Boolean search strings for each database, are provided in Supplementary Table S1 to ensure transparency and reproducibility.

### 2.2. Eligibility Criteria

Eligibility criteria were defined using the PICOS framework.

Population: Studies involving healthy adults, athletic populations, or clinical populations undergoing resistance training or rehabilitation were eligible. Intervention: Blood flow restriction training (BFRT) was selected, either as a standalone intervention or combined with other exercise modalities. Comparators: Active control groups, placebo conditions, traditional resistance training, or other exercise interventions were required. Outcomes: Studies reporting genetic, molecular, physiological, or vascular outcomes relevant to BFRT adaptation were eligible. Primary outcomes were genetic polymorphisms, gene expression, and molecular signalling markers associated with hypertrophy, proteolysis, angiogenesis, and myogenesis. Molecular outcomes of interest included, but were not limited to, IGF-1, MyoD, myogenin, myostatin (MSTN), FOXO3A, Atrogin-1, MuRF-1, VEGF, VEGFR-2, HIF-1α, NOS isoforms, follistatin-related factors, GASP-1, and SMAD-7. Athletic performance was not a primary outcome of interest unless reported alongside BFRT-related genetic, molecular, strength, or hypertrophy outcomes.

Study design: Experimental (RCTs) and quasi-experimental studies, including randomised controlled trials and controlled laboratory studies, were eligible.

Studies were excluded if they involved animal models, did not report relevant BFRT-related outcomes, or were non-original research articles such as reviews, commentaries, and meta-analyses.

### 2.3. Study Selection and Screening Process

Records were imported into Covidence [31] for screening and de-duplication. The titles and abstracts of all remaining articles were independently screened to assess initial relevance based on the predefined inclusion and exclusion criteria derived from the PICOS framework. Articles which passed the initial screening were then reviewed in full text to determine final eligibility. Studies were excluded at this stage if they clearly did not meet the inclusion criteria, such as those testing on animals or studies published outside the 15-year timeframe. This assessment also considered methodological relevance and whether the study provided data linking BFRT outcomes to genetic factors in healthy and athletic populations.

Two reviewers independently screened all titles and abstracts for eligibility. Full-text articles were then assessed independently by both reviewers against the predefined inclusion and exclusion criteria. Any disagreements were resolved through discussion and consensus.

A PRISMA-ScR flow diagram was generated to visually represent the selection process, including the number of studies identified, screened, and assessed for eligibility and ultimately included in the review.

Only three studies met the full inclusion criteria and were included in the final qualitative synthesis.

### 2.4. Methodological Quality Appraisal

The PEDro scale was used to appraise the methodological quality of included randomised controlled trials. Non-randomised and mechanistic studies were not assessed using PEDro because of methodological incompatibility (Table 1).

Alternative appraisal tools such as JBI critical appraisal checklists may have been more appropriate for non-randomised or mechanistic designs and could be considered in future reviews.

### 2.5. Data Extraction

The initial review of studies (Supplementary Table S2) revealed considerable heterogeneity across study designs, including RCTs, systematic reviews, narrative reviews, experimental studies, genetic association studies and meta-analyses. Systematic reviews, narrative reviews, and meta-analyses were screened as part of the broader evidence mapping process but were excluded from the final synthesis of primary BFRT studies.

A structured data extraction table was developed to organise key characteristics of included studies. Data were extracted using a standardised approach to ensure consistency. The following variables were collected: study characteristics (author, year, and study design), participant characteristics (population type and sample size), intervention details (BFRT protocol, load, cuff pressure, and duration), comparator conditions, outcomes measured (molecular, physiological, and performance-related), and key findings relevant to BFRT-induced adaptations. Where reported, information on funding sources and conflicts of interest was also recorded.

Data extraction was conducted independently by two reviewers using the predefined extraction table. Any discrepancies were resolved through discussion and agreement between reviewers.

## 3. Results

### 3.1. Study Selection and Methodological Quality

**Figure 1:**
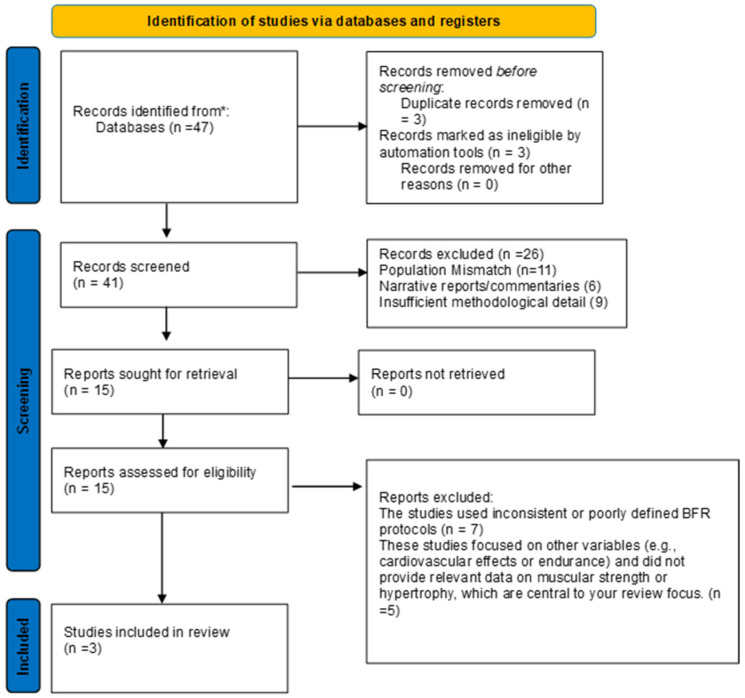
PRISMA-ScR flow diagram of the current study, * = databases investigated, PubMed, Web of Science and Google Scholar.

The study selection process is summarised in Figure 1. The initial search identified 47 records. After removal of duplicate records (*n* = 3) and records removed by automation tools (*n* = 3), 41 records remained for title and abstract screening. Following screening, 15 reports were assessed for full-text eligibility. Of these, three studies met the inclusion criteria and were included in the final qualitative synthesis.

Although both studies reported random allocation, neither described procedures for allocation concealment or assessor blinding, indicating potential risk of selection and detection bias.

### 3.2. Characteristics of Included Studies

Three studies that satisfied the inclusion and exclusion criteria were selected for qualitative synthesis. While these studies did not share common protocols or approaches, together they provide preliminary insight into molecular evidence associated with BFRT-induced adaptations (Table 2).

Two studies [32,33] employed experimental designs involving healthy adult participants. Both investigated the physiological and molecular adaptations to low-load BFR resistance training, with a focus on gene expression related to muscle growth and proteolysis. Larkin et al. [8] examined angiogenic gene expression in response to low-load BFR in a controlled laboratory setting, offering a mechanistic perspective on vascular and metabolic adaptations.

### 3.3. Overview of BFRT Protocols and Outcomes

The three included studies comprised 50 participants in total, with sample sizes ranging from 6 to 29. Two studies involved healthy young adults, while one included healthy older adults. Two studies investigated acute molecular responses to a single BFRT session, whereas one evaluated an 8-week intervention. Training loads ranged from 20% to 40% of one-repetition maximum (1RM). Outcomes included markers of myogenic, proteolytic, and angiogenic gene expression, alongside measures of muscle strength and quadriceps cross-sectional area.

The population varied from healthy, athletic individuals to clinical groups (Table 2 and Supplementary Table S2). This diversity reflects the broad applicability of both BFRT and molecular genetic research in muscular adaptation and performance, whilst highlighting potential differences in physiological responses between groups [3]. Most studies focused on athletes or active individuals aligning with performance-based outcomes of interest, but several studies contributed insights from clinical populations where muscle preservation and rehabilitation were primary goals. Interventions also spanned a wide spectrum. Many studies implemented BFRT protocols, which differed in intensity (low load vs. high load), duration, cuff pressure adjustments and exercise modalities (resistance training or bodyweight training). Others explored electrical stimulation combined with BFR or examined the underlying cellular and molecular mechanisms involved in BFRT. A subset of studies focused solely on genetic polymorphism analysis, investigating the role of specific genes such as *ACTN3*, *MSTN* and *IGF1* in modulating strength, hypertrophy and individual variability in training outcomes, but no studies focused on genetic analysis in conjunction with BFRT. Therefore, only studies examining BFRT-related molecular outcomes could be included in the final synthesis.

The outcomes assessed were equally diverse and often study-specific. Core outcomes included muscle strength and hypertrophy, which are vital for muscular adaptations and are commonly targeted within athletic training programmes. Other studies evaluated performance metrics, muscular damage or fatigue within athletic individuals. Several studies investigated molecular and genetic outcomes, such as myogenic gene expression, signal transduction pathways or the presence of polymorphisms influencing individual responses to training. This range of outcomes reflects both applied and mechanistic approaches to understanding the interplay between BFRT and genetic factors.

### 3.4. Summary of Key Outcomes

**Figure 2:**
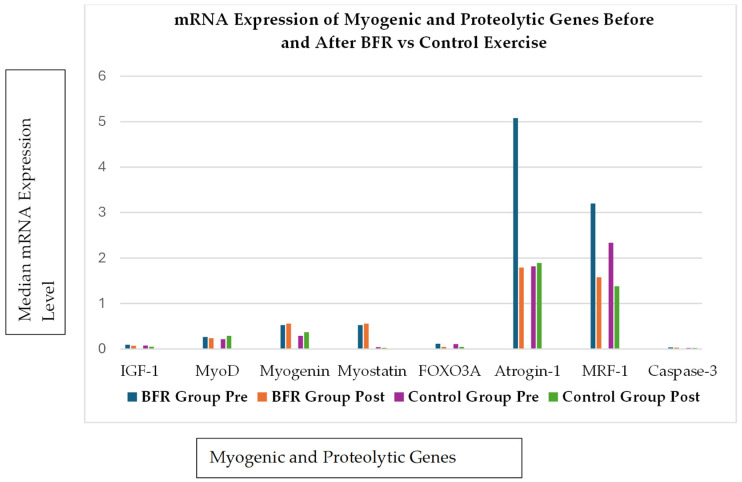
Summary of molecular responses (mRNA expression) associated with muscle growth (*IGF-1*, *MyoD*, *myogenin*, and *myostatin*) and proteolysis (*FOXO3A*, *Atrogin-1*, *MuRF-1*, and Caspase-3) before and after session of blood flow-restricted (BFR) and control resistance exercise. Notably, BFR training was associated with lower expression of proteolytic genes *FOXO3A*, *Atrogin-1*, and *MuRF-1*. Adapted from [32].

**Figure 3:**
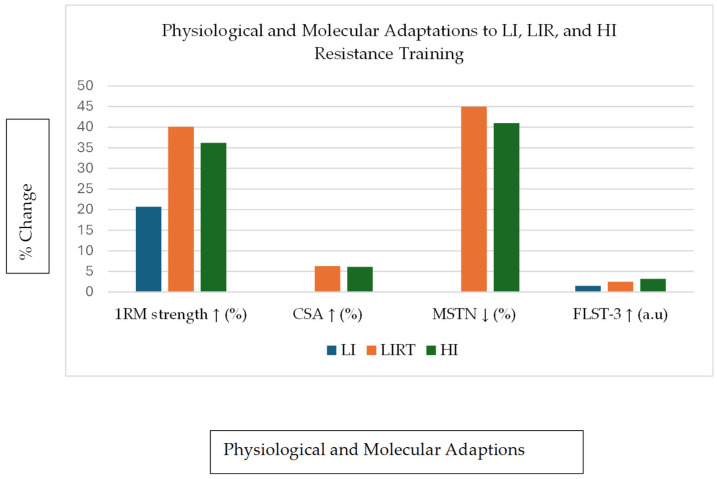
Summary of physiological (1RM and CSA) and molecular (MSTN and FLST-3) responses following 8 weeks of low-intensity (LI), low-intensity with blood flow restriction (LIRT), and high-intensity (HI) resistance training. Redrawn with data from [33].

**Figure 4:**
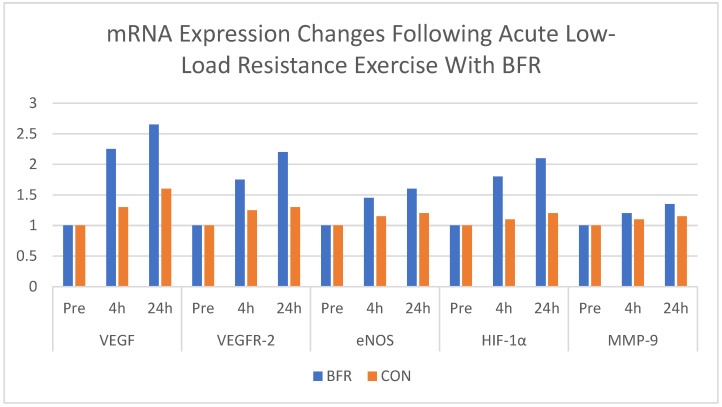
Skeletal muscle mRNA expression (% change Y-axis) of *VEGF*, *VEGFR-2*, *HIF-1α*, *eNOS*, and *MMP-9* before and at baseline (Pre), 4 h and 24 h (X-axis) following acute low-load resistance exercise with and without BFR. Expression is presented as a fold change from baseline. Data are shown as mean values (*n* = 6). Redrawn with data from [8]. BFR = Blood Flow Restriction, CON = Control Group (same exercises without the BFR).

Due to the small number of included studies and substantial heterogeneity, graphical representations are based on individual studies and are presented for illustrative purposes only. No pooled or aggregated analyses were performed. Figure 2, Figure 3 and Figure 4 were prepared by the authors as visual summaries of findings reported in the original studies.

Manini et al. [32] explored the acute molecular responses to LL-RT with BFRT in healthy adults. Participants were randomised into the BFR group or the control group, and they performed four sets of knee extensions at 20% of their 1RM. Muscle biopsies were taken 8 h post-exercise, which revealed no significant changes in the expression of key myogenic genes (*IGF-1*, *MyoD*, myogenin, and myostatin) in either group (Figure 2). However, the BFR group showed a marked downregulation of transcripts involved in proteolysis, specifically *FOXO3A*, *Atrogin-1* and *MuRF-1* genes. These genes are typically associated with muscle protein breakdown.

Laurentino et al. [33] investigated both physiological and molecular adaptations in three training groups (Figure 3). These groups were LI-RT (low-intensity resistance training) with BFR, LI without BFR (low-intensity training) and HI-RT (high-intensity resistance training without BFR). Over 8 weeks, all groups improved muscle strength, with the LI-RT group showing the largest gains (+40.1%). Muscle hypertrophy, measured by the quadriceps cross-sectional area (CSA), was reported to increase in both the low-intensity resistance (LIR) with BFR and the high-intensity resistance (HIR) groups, but not the LI group. On a molecular level, the LI-RT and HI-RT groups demonstrated reductions in myostatin (*MSTN*) mRNA and increases in key anabolic genes such as follistatin (*FLST* and *FLST-3*), *GASP-1* and SMAD-7 (Figure 3). These results were derived from vastus lateralis muscle biopsy samples, indicating local intramuscular rather than systemic adaptations.

Larkin et al. [8] investigated the acute effects of low-load resistance exercise with blood flow restriction on angiogenic gene expression. In a randomised crossover design, six healthy young adults performed unilateral knee extension exercises at 40% 1RM with and without BFR. Muscle biopsies collected at baseline, 4 h, and 24 h post-exercise showed that BFR increased the expression of angiogenesis-related mRNA, including *VEGF*, *VEGF-R2*, *HIF-1α*, *nNOS*, and *iNOS*, compared with the control condition (Figure 4).

Reporting of follow-up assessments, participant dropouts, and adverse events was limited across the included studies. Two studies [32,33] included short-term or intervention-based follow-up periods; however, long-term follow-up data were not reported. Information regarding participant attrition was inconsistently described, and none of the included studies provided detailed reporting of adverse events associated with BFRT. This lack of consistent reporting limits the ability to assess the safety and longer-term effectiveness of BFRT interventions. In addition, the review aimed to identify and map gaps in the literature, particularly regarding genetic polymorphism-based research/analyses. Table 3 highlights considerable variability in BFRT application across studies, particularly in cuff pressure, exercise modality, and intervention duration, which may contribute to differences in reported molecular and physiological responses.

## 4. Discussion

### 4.1. Absence of Genetic Evidence and Interpretation of Findings

**Figure 5:**
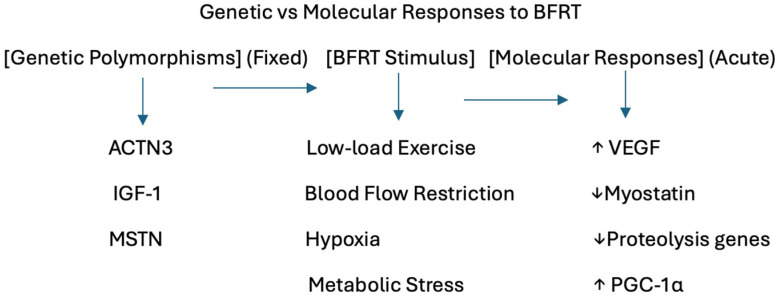
Author-created conceptual illustration distinguishing genetic polymorphisms from acute molecular responses to blood flow restriction training (BFRT).

A central finding of this scoping review is the clear absence of studies investigating genetic polymorphisms in the context of BFRT. This review also highlights the limited number of studies examining molecular responses to blood flow restriction training (BFRT), underscoring the emerging nature of this research area and the need for further research. Notably, no studies have directly examined genetic polymorphisms or genotype-based differences in response to BFRT. Genetic polymorphisms represent inherited, stable traits that may influence baseline responsiveness to exercise [34], while molecular responses reflect acute, exercise-induced signalling pathways activated by BFRT (Figure 5).

These studies suggest that BFRT-induced hypertrophy may involve not only anabolic signalling but also suppression of muscle protein breakdown pathways. Each study provides preliminary insight into distinct mechanisms or outcomes relevant to strength, hypertrophy, and muscular adaptations in athletic populations. No included studies directly assessed genotype-dependent responses to BFRT.

Figure 2 indicates that while markers of myogenesis and anabolic signalling (*IGF-1*, *MyoD*, *myogenin*, and *myostatin*) did not change significantly, BFRT was associated with downregulation of key proteolytic genes (*FOXO3A*, *Atrogin-1*, and *MuRF-1*). This pattern may reflect a shift in the balance between muscle protein synthesis and degradation, whereby attenuation of proteolytic signalling contributes to the hypertrophic response. These findings suggest that BFRT-induced hypertrophy may not depend solely on robust anabolic upregulation but may also involve suppression of muscle protein breakdown pathways [32,33]. Figure 3 reinforces this by showing that BFRT combined with low-intensity resistance training was associated with increases in 1RM strength and muscle CSA comparable to high-intensity training. These functional outcomes coincided with molecular changes, including reductions in myostatin (a negative regulator of muscle growth) and increases in follistatin-like 3 (*FLST-3*), *GASP-1* and *SMAD-7* gene expression. These gene expression profiles are typically associated with muscle growth and regeneration, supporting the possibility that BFRT may elicit hypertrophic responses through molecular mechanisms similar to those observed following high-load training, although this is based on limited evidence [33].

Figure 4 expands on the systemic impact of BFR by investigating its angiogenic effects. The significant increases in VEGF, VEGFR-2, HIF-1α and eNOS gene expression following BFRT indicate that this modality triggers hypoxia-sensitive pathways related to vascular adaptations [8,35]. These findings suggest that BFRT may contribute to vascular adaptations, including changes in blood flow and capillarisation. This combination of muscular and vascular adaptations may provide broader physiological benefits, although direct comparisons with conventional resistance training remain limited. However, some inconsistencies exist in the timing and magnitude of molecular responses. While this study observed significant vascular gene expression following BFR, other studies such as [36] suggest that these responses may be highly transient and influenced by cuff pressure, limb position and training status.

### 4.2. Inconsistencies in Molecular Activation Across Studies

Differences in molecular responses observed between [32] and [33] may be attributed to several key methodological and participant-related variables. One potential factor is participant training status. Ref. [33] implemented an 8-week intervention in resistance-trained individuals, allowing for chronic adaptations to emerge, whereas [32] assessed acute molecular responses in a single-session protocol, which may not reflect long-term gene expression changes. Training history is known to modulate the magnitude and timing of molecular signalling, with trained individuals often exhibiting blunted acute responses compared to untrained individuals [36,37].

Cuff pressure and occlusion duration also varied between studies. Ref. [32] used a standardised pressure but provided limited detail on limb circumference adjustments, whereas [33] applied more individualised pressures, which may have been more effective for inducing metabolic stress and downstream gene expression. Sex-based differences could further explain divergent results; ref. [32] included both male and female participants, while [33] examined an exclusively male cohort. Given that sex hormones modulate myogenic signalling pathways, this may have influenced the expression of genes such as myostatin and follistatin [38].

Taken together, these discrepancies underscore the need for greater standardisation in BFR methodology and consideration of participant characteristics when interpreting molecular outcomes.

### 4.3. Implications for Practice

Beyond performance settings, the ability to induce strength and hypertrophic adaptations using lower external loads has important implications for ageing populations, individuals at risk of sarcopenia and those undergoing musculoskeletal rehabilitation [1,17,18]. The evidence presented in this review suggests that BFRT may offer a practical alternative to traditional high-load resistance training, with implications for a range of professional contexts. In particular, BFRT may provide several comparative advantages, including reduced mechanical load, time efficiency, accessibility due to minimal equipment requirements, and the potential to achieve adaptations comparable to high-load resistance training [39,40].

For strength and conditioning coaches, BFRT can be used strategically during deloading phases or when athletes are recovering from injury but still need to maintain strength and hypertrophy [1]. Its ability to promote muscular adaptations with lighter loads allows for continued progress without excessive joint or tendon stress.

Compared with traditional high-load resistance training, BFRT offers a unique advantage by enabling meaningful strength and hypertrophic adaptations at substantially lower mechanical loads. This is particularly beneficial in situations where high mechanical stress is contraindicated, such as early-stage rehabilitation following musculoskeletal injury.

In clinical and rehabilitation settings, BFR presents a low-impact option for patients unable to tolerate high mechanical loads due to post-operative restrictions or chronic musculoskeletal issues [41,42,43]. Its use can support earlier initiation of resistance-based rehabilitation, potentially accelerating recovery while reducing the risk of re-injury [41,43,44]. Lastly, the relative ease of application, short session durations and minimal equipment requirements make BFRT highly accessible and scalable across various training environments [45,46].

Despite the promising benefits of BFRT, practitioners must approach its use with appropriate caution. Improper cuff pressure, duration or placement may lead to adverse effects such as discomfort, numbness or, in rare cases, vascular complications [36]. Therefore, BFRT should be implemented under guided supervision with evidence-based protocols.

### 4.4. Translational and Public Health Implications

The potential public health relevance of BFRT may be better understood when considered within specific clinical contexts. For example, in ageing populations at risk of sarcopenia, BFRT offers a low-load alternative to traditional resistance training, allowing individuals with reduced strength or mobility limitations to achieve meaningful muscular adaptations. Similarly, in post-operative rehabilitation settings, where high mechanical loads are often contraindicated, BFRT may facilitate earlier engagement in resistance-based exercise while minimising joint stress. Recent evidence also supports the application of BFRT in post-operative rehabilitation following knee surgeries, demonstrating improvements in functional outcomes while maintaining a favourable safety profile, although variability in protocols and outcome measures remains [47,48].

Within these contexts, understanding individual variability in response to BFRT could be clinically valuable. Although no studies to date have directly examined genetic polymorphisms in BFRT settings, it is plausible that genes related to muscle growth (e.g., *MSTN* and *IGF-1*), fibre-type composition (*ACTN3*), and vascular function (NOS3) may influence responsiveness [22,24,26,28]. In the future, such information could potentially be used to refine patient selection or optimise BFRT prescription (e.g., cuff pressure and training intensity), thereby improving intervention effectiveness.

However, from a health systems perspective, the integration of genetic testing into exercise prescription raises important considerations. Issues related to cost, accessibility, and health equity must be carefully balanced against the potential benefits of genotype-informed interventions. The feasibility and clinical value of such approaches therefore remain uncertain and require further investigation before implementation in routine practice can be justified.

### 4.5. Study Limitations

This review was registered retrospectively after completion of the literature search, which reduces transparency compared to prospective protocol registration. Effect sizes and pooled quantitative estimates were not calculated because of the small number of included studies, heterogeneity in design and outcomes, and inconsistent reporting of effect estimates in the original studies.

While the findings presented in this review highlight the interactions between BFRT and molecular adaptations linked to muscle hypertrophy, strength and genetic modulation, several limitations must be acknowledged.

Firstly, the number of studies examining BFRT alongside molecular responses is limited, with no studies directly assessing genetic polymorphisms. The sample sizes in the included studies were relatively small, varying from 6 to 29 participants [8,32,33], which limits statistical power and generalisability. Most studies also relied on young, healthy participants, often male, therefore reducing the applicability of findings to female athletic populations.

Secondly, the duration and design of the studies varied considerably. For example, ref. [33] employed an 8-week training programme with physiological and molecular outcome measures, whereas refs. [8,32] focused on acute post-exercise molecular responses. This heterogeneity in the study duration, outcome focus (acute vs. chronic) and methodological protocols complicates direct comparison and synthesis. It also makes it difficult to determine whether observed molecular changes translate into long-term structural or performance adaptations. Standardising intervention length and combining acute molecular markers with chronic performance outcomes in future studies would strengthen the evidence base and improve comparability across trials.

Thirdly, there was a lack of consistent reporting regarding safety, adverse events, and long-term outcomes. This is particularly important given the increasing application of BFRT in clinical and public health contexts, where safety and tolerability are critical considerations.

Collectively, the physiological and molecular adaptations observed across the BFRT literature provide preliminary support for its application in both athletic and rehabilitative contexts. While physiological and vascular adaptations have been demonstrated, the extent to which polymorphisms moderate these responses is unknown. This highlights an important gap in the literature: adaptations to BFRT are well established, but the role of genotype in explaining individual variability in BFRT adaptations has yet to be directly investigated.

There is a potential for publication bias within this emerging field, with studies more likely to report positive or significant mechanistic findings. This may lead to an overestimation of the effectiveness or consistency of BFRT-induced molecular adaptations.

Furthermore, several included studies incorporated variations in training protocols or additional stimuli alongside BFRT, which may act as concomitant interventions and confound the interpretation of BFRT-specific effects. This makes it difficult to isolate the independent contribution of BFRT to the observed molecular and physiological outcomes. In addition, without accompanying protein or performance data, the interpretation of molecular signalling as predictive of actual training effect remains limited.

### 4.6. Suggestions for Future Research

Future research should explore the dose–response relationship of key BFR variables. Examples of this would include: cuff pressure, occlusion duration, training intensity and molecular as well as physiological outcomes. All studies included in this review utilised BFR protocols; a lack of standardisation limits comparability. For instance, refs. [8,33] used different BFR pressures and training volumes, which may have influenced muscle adaptations. Controlled studies that systematically vary BFR parameters would help establish optimal protocols for specific goals, such as hypertrophy compared with endurance.

Also, there is a need to examine longitudinal gene expression responses in BFR training. While studies like [8,32] provide valuable insight into acute molecular responses, it remains unclear how these transient changes translate into long-term adaptations. Future studies should include multiple time points over extended training periods to track the persistence of gene expression changes and their relationship to muscle growth and strength.

Future studies should also expand beyond small, homogeneous cohorts by incorporating data from larger sports and exercise physiology databases. This would allow researchers to explore how BFRT adaptations may vary across sex, age, sporting background and geographical location, factors which are likely to interact with genetic variation [38]. Future investigations must shift from theoretical associations to experimentally verified relationships. Carefully controlled, large multi-centre investigations should standardise BFRT protocols, stratify or randomise by genotype, and integrate molecular (mRNA/protein), morphological CSA and performance endpoints over meaningful training durations. Multi-centre trials drawing on international cohorts could therefore strengthen the current knowledge and improve the generalisability of findings.

Interdisciplinary studies that integrate genetic, molecular and functional performance measures would provide a more holistic view of individual responses to BFR. With the rise in personalised training, research should focus on identifying molecular or genetic biomarkers that predict who may benefit most from BFR interventions, thereby improving programme design in both athletic and clinical settings.

Importantly, the included studies assessed acute molecular responses rather than genotype-dependent adaptations, reinforcing the distinction between molecular signalling and fixed genetic traits. However, at present, genotype-guided BFRT prescription is not supported by empirical evidence.

Given the small number of included studies and substantial heterogeneity in cuff pressure, load, and intervention duration, the current evidence is insufficient to propose a baseline BFRT dosage for activation of molecular or genetic pathways.

## 5. Conclusions

This scoping review identified very limited evidence on genetic and molecular modulators of adaptation to blood flow restriction training (BFRT). The principal finding of this review is the complete absence of studies investigating genetic polymorphisms in relation to BFRT adaptations. Only three eligible studies were identified, and none directly examined genetic polymorphisms or genotype-dependent responses to BFRT. The available evidence, therefore, reflects molecular signalling responses rather than inherited genetic influences on adaptation. Although BFRT was associated with reported changes in proteolytic, myostatin-related, and angiogenic pathways, the small number of studies and considerable methodological heterogeneity limit the strength of conclusions. At present, genotype-informed BFRT prescription remains premature. Future research should prioritise adequately powered, prospective studies integrating genetic, molecular, morphological, and functional outcomes using standardised BFRT protocols.

## Figures and Tables

**Table 1:** Methodological quality appraisal of included randomised controlled trials using the PEDro scale.

Number	Criterion	Manini et al., 2011 [32]	Laurentino et al., 2012 [33]
1	Eligibility criteria	Yes	Yes
2	Random allocation	Yes	Yes
3	Concealed allocation	No	No
4	Baseline comparability	No	No
5	Blinding of participants	No	No
6	Blinding of therapists	No	No
7	Blinding of assessors	No	No
8	>85% follow-up	Yes	Yes
9	Intention to treat	No	No
10	Between-group statistical comparisons	Yes	Yes
11	Point estimates and variability	Yes	Yes
	PEDro total score	4/10	4/10
	Sample size ≥ 50	No	No
	Level of evidence	Level II	Level II

Item 1 is not included in the total PEDro score.

**Table 2:** Characteristics and key findings of included studies.

Study	Design	Population	Sample Size	Comparator	Outcomes	Key Findings
Manini et al., 2011 [32]	RCT	Healthy adults	*n* = 15	Low-load resistance training (LL-RT)	mRNA expression (IGF-1, MyoD, myogenin, myostatin), proteolytic genes (FOXO3A, Atrogin-1, MuRF-1)	BFRT was associated with downregulation of proteolytic gene expression, with no change in anabolic gene expression
Laurentino et al., 2012 [33]	RCT	Healthy males	*n* = 29	LL-RT and high-load resistance training (HL-RT)	Strength (1RM), quadriceps cross-sectional area (CSA), myostatin (MSTN), anabolic gene expression	BFRT was associated with increases in strength and CSA, reductions in myostatin expression, and increases in anabolic gene expression, comparable to HL-RT
Larkin et al., 2012 [8]	Non-randomised crossover experimental study	Healthy young adults	*n* = 6	Exercise without BFR	Angiogenic mRNA expression (VEGF, VEGFR-2, HIF-1α, NOS)	BFRT was associated with increased expression of angiogenic genes following acute exercise

**Table 3:** Summary of BFRT protocol characteristics across included studies.

Study	BFR Pressure	Exercise Load	Duration	Session	BFR Device	Concomitant Interventions
Manini et al., 2011 [32]	Not clearly reported	20–30% 1RM	Acute (single session)	1 session	Pneumatic Cuff	None
Laurentino et al., 2012 [33]	Not clearly reported	20% 1RM (BFR) 80% 1RM (HI)	8 weeks	Multiple sessions per week	Pneumatic Cuff	Resistance Training
Larkin et al., 2012 [8]	220 mmHg	40% 1RM	Acute	1 session	Kaatsu-Master	None

## Data Availability

No new data was created in this analysis and review.

## Supplementary Materials

The following supporting information can be downloaded at: https://www.mdpi.com/article/10.3390/ijerph23050567/s1, Table S1: Search strategies for different searches/databases; Table S2: Initial list of reviewed papers and their attributes according to PICOS guidelines.

## Abbreviations

BFR	Blood Flow Restriction
BFRT	Blood Flow Restriction Training
CSA	Cross-sectional Area
HIF-1α	Hypoxia-inducible Factor 1 Alpha
IGF-1	Insulin-like Growth Factor 1
mRNA	Messenger Ribonucleic Acid
MSTN	Myostatin
RCT	Randomised Controlled Trial
VEGF	Vascular Endothelial Growth Factor
