# Supplementary material for: Blood Flow Restriction Training, Molecular Modulators, and Musculoskeletal Health: A Scoping Review and Translational Perspective
Source: Int J Environ Res Public Health. 2026 Apr 28;23(5):567. doi: 10.3390/ijerph23050567 (PMC13207192; doi:10.3390/ijerph23050567)
Supplement: Supplementary file 1 [file ijerph-23-00567-s001.zip › ijerph-4199924-supplementary.pdf]

Supplementary Table S1: Search strategies for different searches/databases

A1.PubMed Search Strategy

("blood flow restriction" OR "BFR training" OR "blood flow restriction training" OR "vascular occlusion")
AND
("muscle strength" OR "strength" OR "muscle hypertrophy" OR "muscle growth" OR "muscle size" OR "muscle mass")
AND
("genetic" OR "genetics" OR "gene" OR "polymorphism" OR "genotype" OR "genetic variation" OR "genetic predisposition")
AND
("athletic" OR "athlete" OR "healthy" OR "sportspeople" OR "sportsperson")

A2. Web of Science Search Strategy

("blood flow restriction" OR "BFR training" OR "vascular occlusion")
AND
("muscle strength" OR "muscle hypertrophy" OR "muscle growth" OR "muscle mass")
AND
("genetic" OR "gene" OR "polymorphism" OR "genotype" OR "genetic variation")
AND
("athlete" OR "healthy" OR "sport")

A3. Google Scholar Search Strategy

"blood flow restriction" AND muscle AND (gene OR genetic OR polymorphism)

Supplementary Table S2: Initial list of reviewed papers and their attributes according to PICOS guidelines

**Study Reference**	**Study Design**	**Population**	**Intervention**	**Outcomes**	**Geneti c Focus**	**Include d in Results**
Lorenzo et al., 2021 [1]	Systematic Review	Athletes	BFRT	Increased strength and hypertrophy	No	No
Schwiete et al., 2025 [2]	Review	Various	Muscle fatigue/damag e	Continuum concept	No	No
Early et al., 2020 [3]	RCT	Healthy adults	BFR training	Performance, pain	No	No
Saatmann et al., 2021 [4]	Meta-analysis	Type 2 diabetics	BFR therapy	Low oxygen tension during BFR	No	No
Vopat et al., 2019 [5]	Review	Athletes	BFR therapy	Application and future	No	No
Cerqueira et al., 2021 [6]	Experimenta l	Healthy adults	BFR with adjusted cuff pressure	Muscle adaptation	No	No
Larkin et al., 2012 [8]	Experimenta l	Athletes	Low-load BFR	Angiogenic gene expression	Yes	Yes
Chu et al., 2025 [9]	Experimenta l	Rats (model)	Electrical stimulation + BFR	Muscle repair	Indirec t	No
Martin et al., 2022 [10]	Review	Various	BFR overview	Muscle adaptation	No	No
Dankel et al., 2016 [15]	Experimenta l	Healthy individuals	BFR effects on muscle sites	Muscle size	No	No
Wortman et al., 2020 [17]	Systematic Review	Athletes	BFR in athletes	Performance	No	No
Geng et al., 2024 [18]	Meta-analysis	Mixed population s	BFR vs high-load training	Strength & hypertrophy	No	No

Castilla-López & Romero-Franco, 2023 [19]	RCT	Pro soccer players	Low-load BFR vs high-load training	Performance	No	No
Pickering & Kiely, 2017 [20]	Review	Various	ACTN3 gene	Performance	Yes	No
Kim, Song & Kim, 2014 [21]	Experimental	Athletes	Comparing distribution of ACTN3 genotypes and alleles	Only the speed-oriented athletes showed significant differences in the frequency distributions of the ACTN3 XX genotype	Yes	No
El Ouali et al., 2024 [23]	Meta-analysis	Athletes	ACTN3 polymorphism	Performance	Yes	No
Yoshida & Delafontaine, 2020 [24]	Review	Various	IGF-1 pathway	Hypertrophy/atrophy	Yes	No
Mendes et al., 2024 [25]	Meta-analysis	Athletes	IGF1 polymorphism	Athletic performance	Yes	No
Leońska-Duniec et al., 2023 [26]	Genetic Association Study	Elite athletes	Genetic variants	Athletic status	Yes	No
Kruszewski & Aksenov, 2022 [27]	Systematic Review	Athletes	MSTN gene polymorphisms	Strength	Yes	No
Manini et al., 2011 [32]	Experimental	Older adults	BFR resistance training	Myogenic mRNA expression	Yes	Yes
Laurentino et al., 2012 [33]	Experimental	Young males	BFR resistance training	Myostatin gene expression	Yes	Yes
Ramakrishnan et al., 2015 [35]	Review	Various	Vascular endothelial growth factor	VEGF-based therapies	No	No
Murphy, 2025 [45]	Systematic Review	Healthy adults	BFRT	Hypertrophy & strength	No	No

Legend

- *BFR = Blood Flow Restriction*
- *BFRT = Blood Flow Restriction Training*
- *RCT = Randomized Controlled Trial*
- *CV = Cardiovascular*
- *IGF1 = Insulin-like Growth Factor 1*
- *ACTN3 = Alpha-Actinin-3 gene*
- *VEGF = Vascular Endothelial Growth Factor*
- *AAS = Anabolic Androgenic Steroid*
- *mRNA = Messenger Ribonucleic Acid*
- *MSTN = Myostatin gene*
